# Supplementary material for: Inflammatory Skin Disease Causes Anxiety Symptoms Leading to an Irreversible Course
Source: Int J Mol Sci. 2023 Mar 21;24(6):5942. doi: 10.3390/ijms24065942 (PMC10058663; doi:10.3390/ijms24065942)
Supplement: Supplementary file 1 [file ijms-24-05942-s001.zip › ijms-2197844-supplementary.pdf]

Supplemental Figure

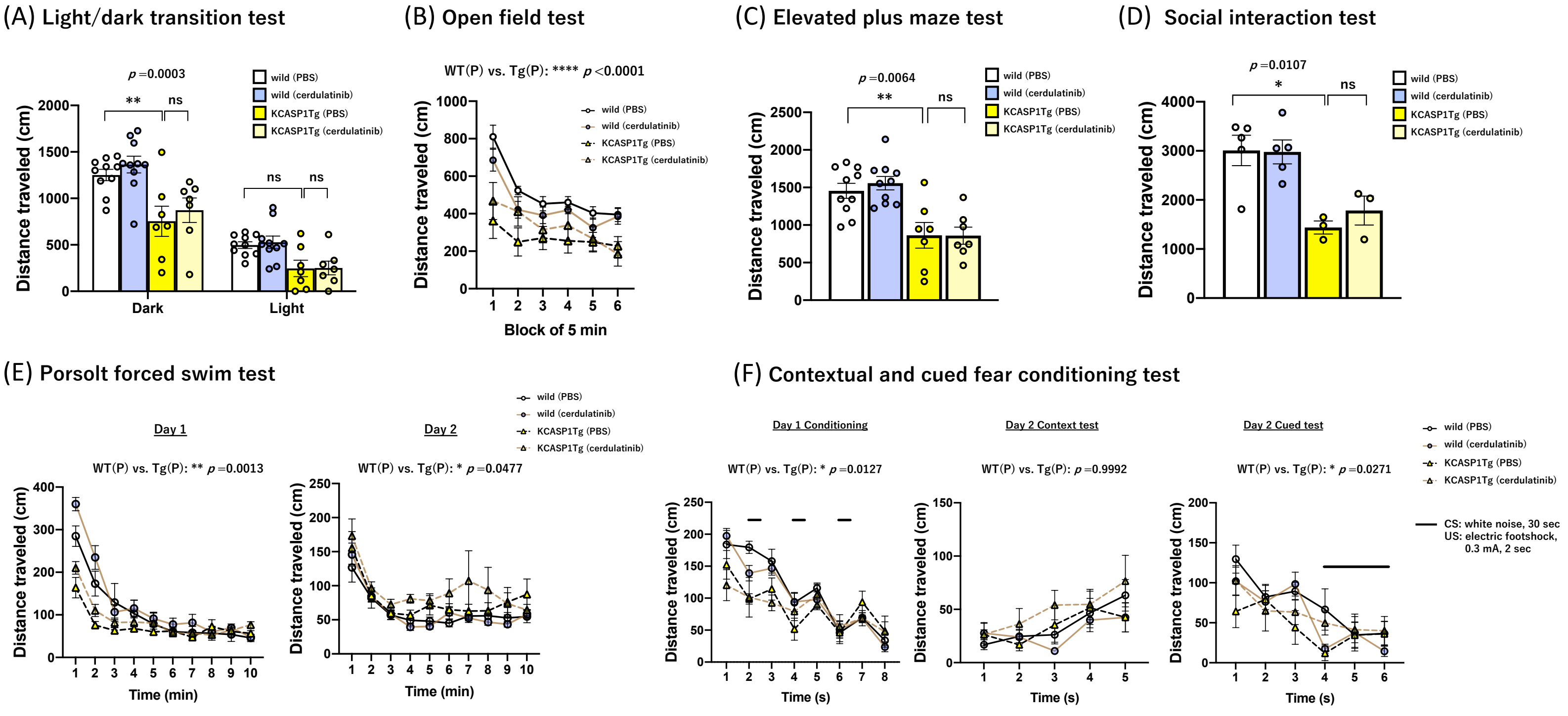

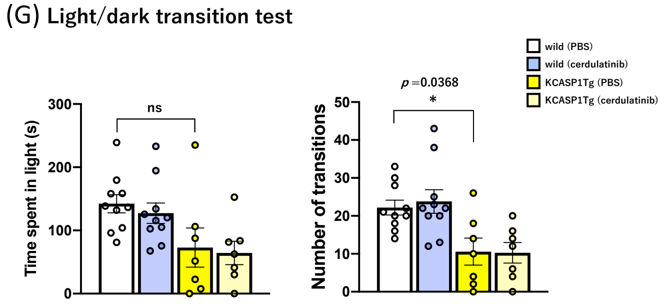

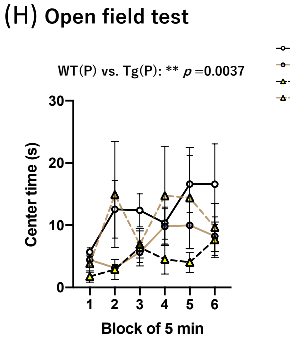

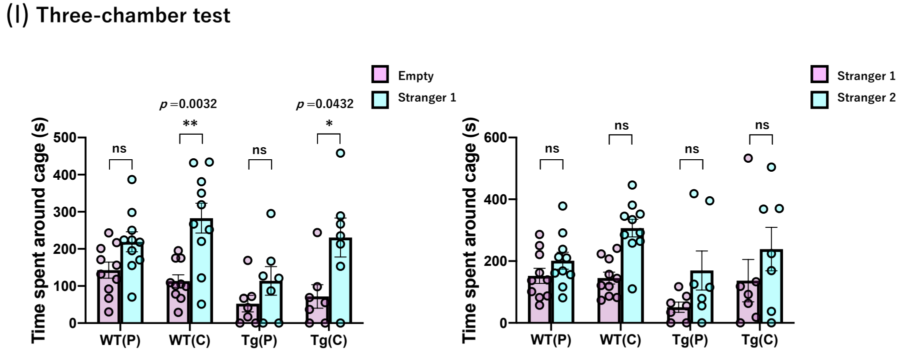

(A-F) KCASP1Tg mice showed hypoactivity in LD, light/dark transition test; OF, open field test; EP,elevated plus maze test; SI, social interaction test; PS, porsolt forced swim test and FZ, contextual and cued fear conditioning test. (G,H) KCASP1Tg mice were suggested to have anxiety tendency in LD and OF. (I) KCASP1Tg mice were suggested to decrease social interest in CSI, three-chamber test. All data are expressed as the mean ± SEM by ordinary one-way or two-way ANOVA, followed by Tukey's multiple comparison test (* $p < 0.05$, ** $p < 0.01$, **** $p < 0.0001$, ns: not significant.).
